# Effects of Surface Protein Adsorption on the Distribution and Retention of Intratumorally Administered Gold Nanoparticles

**DOI:** 10.3390/pharmaceutics13020216

**Published:** 2021-02-05

**Authors:** Rossana Terracciano, Aobo Zhang, E. Brian Butler, Danilo Demarchi, Jason H. Hafner, Alessandro Grattoni, Carly S. Filgueira

**Affiliations:** 1 Department of Nanomedicine, Houston Methodist Research Institute, Houston, TX 77030, USA; rterracciano@houstonmethodist.org (R.T.); agrattoni@houstonmethodist.org (A.G.); 2 Department of Electronics, Politecnico di Torino, 10129 Torino, Italy; danilo.demarchi@polito.it; 3 Department of Physics & Astronomy, Rice University, Houston, TX 77005, USA; aobo.zhang@rice.edu (A.Z.); hafner@rice.edu (J.H.H.); 4 Department of Radiation Oncology, Houston Methodist Research Institute, Houston, TX 77030, USA; ebutler@houstonmethodist.org; 5 Department of Chemistry, Rice University, Houston, TX 77005, USA; 6 Department of Surgery, Houston Methodist Research Institute, Houston, TX 77030, USA; 7 Department of Cardiovascular Surgery, Houston Methodist Research Institute, Houston, TX 77030, USA

**Keywords:** gold nanoparticles, theranostics, in vivo computed tomography imaging, non-small cell lung cancer, in vivo biodistribution, surface passivation, inductively coupled plasma optical emission spectrometry

## Abstract

The heterogeneous distribution of delivery or treatment modalities within the tumor mass is a crucial limiting factor for a vast range of theranostic applications. Understanding the interactions between a nanomaterial and the tumor microenvironment will help to overcome challenges associated with tumor heterogeneity, as well as the clinical translation of nanotheranostic materials. This study aims to evaluate the influence of protein surface adsorption on gold nanoparticle (GNP) biodistribution using high-resolution computed tomography (CT) preclinical imaging in C57BL/6 mice harboring Lewis lung carcinoma (LLC) tumors. LLC provides a valuable model for study due to its highly heterogenous nature, which makes drug delivery to the tumor challenging. By controlling the adsorption of proteins on the GNP surface, we hypothesize that we can influence the intratumoral distribution pattern and particle retention. We performed an in vitro study to evaluate the uptake of GNPs by LLC cells and an in vivo study to assess and quantify the GNP biodistribution by injecting concentrated GNPs citrate-stabilized or passivated with bovine serum albumin (BSA) intratumorally into LLC solid tumors. Quantitative CT and inductively coupled plasma optical emission spectrometry (ICP-OES) results both confirm the presence of particles in the tumor 9 days post-injection (*n* = 8 mice/group). A significant difference is highlighted between citrate-GNP and BSA-GNP groups (** *p* < 0.005, Tukey’s multiple comparisons test), confirming that the protein corona of GNPs modifies intratumoral distribution and retention of the particles. In conclusion, our investigations show that the surface passivation of GNPs influences the mechanism of cellular uptake and intratumoral distribution in vivo, highlighting the spatial heterogeneity of the solid tumor.

## 1. Introduction

Theranostic nanomedicine for cancer management offers innovative strategies to non-invasively detect and diagnose the disease at its earliest premalignant state, and to provide specific therapy against its progression and reoccurrence [1,2]. However, one of the most significant challenges associated with the translation of theranostic nanomedicine to the clinic is the interaction between the nanomaterial and the tumor microenvironment [3]. In particular, when nanoparticles enter a biological system, their interaction with proteins can lead to the formation of a protein corona adsorbed on their surface via electrostatic, hydrophobic, and van der Waals forces [4], which can alter particle stability [5,6], dispersibility [7,8], biodistribution [9], pharmacokinetics [10,11,12], and the toxicity profile [13,14,15].

Due to their unique optical properties [16,17,18], combined with their high biocompatibility and lack of toxicity [19,20,21], gold nanoparticles (GNPs) have demonstrated success among nanotheranostic cancer-related applications [22]. In addition to solid gold particles of various shapes and dimensions (nanobelts [23], nanowires [24], nanostars [25], etc.), core-shell gold-coated particles [26,27,28] have also been rationally designed for application in cancer therapies [29,30]. However, successful in vivo outcomes of the use of GNPs are strongly dependent on the interactions between the protein corona layer and the surrounding cells [31,32]. Understanding GNP-protein interactions is crucial for the development, manufacturing, and translation of GNP-based nanotheranostics [33,34]. An extensive body of literature has shown the effects of the surface chemistry and size of spherical GNPs on the protein corona, with the aim of controlling opsonization on GNPs [35,36,37].

The protein corona formed around the particle when administered in vivo is composed of a complex range of adsorbed proteins, such as albumin, immunoglobulin, glycoproteins, and apolipoproteins [38], which are proteins of lower affinity and higher abundance that bind initially, and over time, are replaced by higher affinity proteins, such as fibrinogen or lysozyme [39]. There are mainly two layers of proteins: an inner layer of irreversibly bonded proteins interacting directly with the GNP surface, which is called the hard corona, and an outer layer of proteins linked through weak protein-protein interactions, called the soft corona [40]. The displacement of the hydration layer which leads to the formation of the overall particle corona is a complex, dynamic, and competitive process for stabilizing the GNPs in a physiological environment [41]. In this configuration, epitopes which are normally buried in the interior sites of proteins can be exposed outwards from the soft corona layer of the particle [42], making GNPs recognizable for phagocytes [43], and consequently causing the rapid clearance of the nanoparticles from plasma, as well as accumulation in the liver and spleen [44]. Understanding how to control the physiological properties of GNPs can help mediate processes, such as cellular uptake [45], immunological response [46], toxicity [47], circulation time [48], and transport from one organ to another, as well as their clearance [49]. While many studies focus on the protein content of blood and harnessing the enhanced permeability and retention effect (EPR) for particle accumulation near the tumor after systemic administration, similar principles related to the protein absorption can be considered for intratumoral injection, since other biological compartments of the body, such as the interstitial fluid of tumors, also contain a high protein content [50] that can affect particle behavior. Therefore, the mechanisms studied, related to the formation of a protein corona from the hematic system, can also be applied for other fluids.

In this study, we aim to evaluate the influence of protein surface adsorption on GNP in vivo biodistribution and retention after intratumoral injection. To study our nanoparticle conjugates, we used a non-small-cell lung cancer (NSCLC) murine model, because its heterogeneity involves not only cancer cells but also tumor-infiltrating cells, as well as the surrounding microenvironment [51]. Lung cancers and other solid tumors also contain stromal cells, such as fibroblasts and endothelial cells. Further, LLC tumors are considered highly heterogeneous as they contain subpopulations of cells of widely differing metastatic potentials [52]. Tumor heterogeneity is an important cause of therapy resistance due to non-uniform drug distribution [53]. We hypothesize that by controlling the adsorption of proteins on the GNP surface, we can modulate the zonal distribution of the particles in the tumor. We previously demonstrated that our spherical GNPs have a significant radio-sensitization property in vitro [54], inducing DNA damage in Lewis lung carcinoma (LLC) cells, as well as excellent properties as contrast agents for computed tomography (CT) in vivo [55]. However, these preliminary studies did not consider the hypothesis that surface protein adsorption can affect the intratumoral distribution and retention of the particles. Therefore, in this work, we exploit CT imaging as a non-invasive pre-clinical method to monitor and quantify the biodistribution of functionalized GNPs and highlight the differences in terms of spatial heterogeneity modulated by surface passivation.

## 2. Materials and Methods

### 2.1. Synthesis of Gold Nanoparticles

Spherical GNPs were fabricated using citric acid (Sigma, St. Louis, MO, USA, C3674) rapidly combined with gold (III) chloride (Sigma, St. Louis, MO, USA, 379948). To achieve particle synthesis, an Erlenmeyer flask containing 600 μL of MilliQ water was allowed to boil using a heating mantle. After 30 s of refluxing in the flask, 4.8 mL of 0.039 M aqueous citrate was combined using a serological pipette. While continuing to boil, 7 mL of 0.033 M gold (III) chloride was next added in a single continuous motion, and the solution was left undisturbed as the color gradually transitioned from yellow to black to the final dark red. After room temperature equilibration, the pH of the solution was measured (pH = 3.5), and the sample stored for further use. This protocol results in the synthesis of citrate-stabilized GNPs in the size range of 30–40 nm. The average mean size and error per batch was measured by obtaining electron microscopy images of the sample and importing the images into Matlab for analysis (see Section 2.3). The pH of the solution was adjusted up to 6 using drop-by-drop addition of a 1 M NaOH solution. GNPs (pH = 6) were centrifuged at 1500× *g* for 5 min with an Eppendorf Centrifuge 5810R (Hamburg, Germany) using Amicon Ultra-15 100K filters purchased from Sigma-Aldrich (St. Louis, MO, USA, UFC910008). The filtrate solution (the water filtered from centrifugation) was stored for future dilutions and functionalization. Particles were concentrated up to 10 mg/mL, and stored at 4 °C.

### 2.2. Surface Passivation of Gold Nanoparticles

We selected bovine serum albumin (BSA, molecular weight 66.5 kDa) as a protein model since it is a soluble constituent of blood plasma and, therefore, it can be considered suitable for in vivo investigations. BSA powder (Sigma, St. Louis, MO, USA, A4503) was dissolved in the filtered water after particle centrifugation to obtain a 1 mg/mL solution. Only freshly prepared BSA solutions were used in these experiments and pH was monitored and maintained. Different volumes taken from this initial stock solution were added to the concentrated GNPs and allowed to incubate for 1 h at room temperature for surface passivation (See Section 2.4.1). The solution was stored at 4 °C.

### 2.3. Characterization and Physicochemical Properties of Gold Nanoparticles

Fabricated GNPs were characterized with spectroscopy using a UV/Vis scanning spectrophotometer (DU 730, Beckman Coulter, Inc., Brea, CA, USA). Typically, the UV−Vis spectrum of spherical non-aggregated GNPs has a band around 530 nm, due to the surface plasmon resonance (SPR), plus an absorption edge at shorter wavelengths due to inter-band transitions of d-band electrons. Dynamic light scattering (DLS) offered an analytical means to determine the particle size and polydispersity index (PDI), and Zeta Potential measurements were obtained using a Zetasizer Nano ZS (Malvern Panalytical, MA, USA). For DLS, the sample at a concentration below 1 mg/mL and at a volume of 1 mL was placed in a four-sided cuvette and measured at 25 °C. For this technique, the Brownian motion is measured and related to particle size by illuminating the particles with a laser and analyzing the intensity fluctuations in the scattered light to report a mean size. For Zeta Potential, 1 mL of particle sample was placed in a four-sided cuvette capped by the universal dip cell ZEN1002. For this technique, the instrument determines the electrophoretic mobility by performing an electrophoresis experiment on the sample and measures the velocity of the particles using laser Doppler velocimetry. Particles were imaged with an FEI Nova NanoSEM 230 (FEI Co., Hillsboro, OR, USA) under STEM mode with the vacuum set to 15 KV for both bright and dark field and measured using Matlab (v9.9.0.1467703, R2020b, The MathWorks, Inc., Natick, MA, USA). Surface-enhanced Raman scattering (SERS) spectra of 3 μM BSA-GNPs were obtained using a custom Raman microspectrometer [56] with a 785 nm wavelength light source to calculate Raman shift values and tentative band assignments due to particle passivation. The sample was measured in a capillary tube where the beam spot was focused through an objective 50 μm past the glass/solution interface. An unenhanced Raman spectrum of BSA solution was recorded and subtracted from the BSA-GNPs SERS spectrum to remove any unenhanced Raman contributions.

GNP solutions in concentrations in the range 0–10 mg/mL were aliquoted into microcentrifuge tubes and imaged using a Siemens Inveon High-Resolution Micro-CT to assess their CT contrast properties and compare with a standard contrast agent (Omnipaque™ iohexol for injection 350 mg I/mL, GE Healthcare, Chicago, IL, USA). To avoid concentration gradients that can be created by larger particle sediments, samples were vortexed immediately before imaging. The CT parameters were a slice thickness of 105 μm, in a plane resolution of 105 μm, tube voltage at 80 kV, tube current at 500 μA, and exposure time of 240 ms. X-ray attenuation intensity was calculated in a Hounsfield unit (HU) by processing the digital CT images (DICOM files) using a 3DSlicer (v.4.11.0, open source software, accessible at www.slicer.org) [57]. Quantification analysis was performed by using 3DSlicer and selecting a 3D reconstructed region of interest (ROI) for each sample and then recording the mean attenuation value and plotting as a function of gold and/or iodine concentration in mg/mL. Weber contrast was calculated using the Equation (1):

(1) $$W_{c}=\frac{I\;-{\;I}_{T}\;}{I_{T}}\;\times 100$$

where I is the attenuation value (HU) of a tumor ROI after GNPs/contrast injection and I_T_ is the attenuation value (HU) of the tumor baseline. GNP concentration was determined applying the Beer-Lambert Law on the SPR peak calculated by UV-Vis spectroscopy, assuming that the particles are spherical. Concentrations were also confirmed by inductively coupled plasma optical emission spectrometry (ICP-OES).

### 2.4. BSA Adsorption on Gold Nanoparticles

The interaction of proteins with GNPs depends on variables, such as the chemistry of the adsorbed material and the medium components [58]. In this section, we investigate the nature and concentration of the BSA to be adsorbed, and its relationship with the pH of the immobilization medium.

#### 2.4.1. Preparation of BSA-GNP Conjugates at Different pH Values

As the pH of the medium increases, the sorption properties of the GNPs change, generating a transition of monolayer protein immobilization to multilayers. Sotnikov et al. [59] demonstrated that the pH of the immobilization medium can effect protein adsorption on GNPs: as the pH is modified from 4–5 to 8–10, an increase occurs in the maximum amount of adsorbed protein molecules on a GNP surface, likely due to this protein immobilization layer transition. In alkaline solutions, however, the GNP surface is not fully saturated, so interactions can occur between the BSA-GNPs and other proteins in the body, which can alter the protein corona structure, consequently also changing the proprieties of the particles. However, our interests are more focused towards application in a slightly acidic environment, such as the tumor environment [60]. To determine the saturation of the GNPs surface and protein layer, the GNP solution was adjusted to a desired pH, as described in Section 2.2. BSA solution was then added to the centrifuged GNP solutions to reach a final concentration ranging from 0.5 to 15 μM. pH values for these studies were maintained at 4.7, 6, 7, and 8.5. All experiments were conducted at ambient room temperature.

#### 2.4.2. Adsorption Model

The adsorption model adopted here follows the work from Dominguez-Medina et al. [61] and Röcker et al. [62]. We approximated a BSA molecule as an equilateral triangular prism with height 3.4 nm, and the GNP as a sphere with hydrodynamic radius (R) obtained by DLS measurement before surface passivation. Therefore, an increase in height below 2 × 3.4 nm corresponds to no more than a single layer adsorption of BSA on the spherical GNP surface. The dependence of the hydrodynamic radius r([BSA]) on the number of protein molecules bound to a spherical GNP, assuming that the protein-coated nanoparticle can still be approximated by a sphere, is expressed by the following Equation (2) from. [61]:

(2) $$r\left(\left[\mathrm{BSA}\right]\right)=R\;\sqrt[{3}]{1+\frac{V^{\mathrm{BSA}}}{V^{\mathrm{GNP}}}\frac{N}{1+{\left(\frac{K}{\left[\mathrm{BSA}\right]}\right)}^{n}}}$$

where N represents the average number of protein molecules bound to the nanoparticles at a specific BSA concentration in the solution, *n* is the Hill coefficient (unitless), and K (mol/L) is the dissociation coefficient, which quantifies the strength of the protein-nanoparticle interaction; $V^{\mathrm{GNP}}$ is the volume of the uncoated particles (L), and $V^{\mathrm{BSA}}$ is the molecular volume (L) of the bound protein (BSA). The experiment was performed by adding BSA solutions in different concentrations to the GNPs. The concentration of gold was kept constant, while the BSA concentration was variable. No aggregation or flocculation occurred (confirmed by UV-VIS spectroscopy). DLS measurements were performed on each sample in triplicate, and the hydrodynamic radius (Z-average divided by 2) was calculated. Data were analyzed by fitting an adsorption isotherm over the considered range of BSA concentrations using the modified Langmuir model (Equation (1)) and standard Langmuir model (Equation (1), *n* = 1). Data fitting was performed with Matlab (lsqnonlin, v9.9.0.1467703, R2020b, The MathWorks, Inc., Natick, MA, USA).

#### 2.4.3. Characterization in Various Media

To investigate changes in the effect of BSA adsorption on the GNP protein corona, particles were dispersed in media representing different sources of protein. We tested the particles dispersed in either 600 μM BSA-enriched PBS, plasma obtained from healthy porcine (Male Castrated Yucatan Minipig, ~38 kg, S&S Farms, Ramona, CA, USA) approved by the Institutional Animal Care and Use Committee (IACUC) at the Houston Methodist Research Institute (approved code: AUP-0620-0035, 2 June 2020), or fetal bovine serum (FBS) (ATCC, Manassas, VA, USA, 30-2020). To obtain plasma, whole blood was collected in EDTA-treated tubes and centrifuged for 15 min at 2000× *g*. We performed DLS measurements to calculate the hydrodynamic diameter and UV-Vis spectroscopy as described in Section 2.3.

### 2.5. Cellular Uptake of GNPs and Cytotoxicity In Vitro

Since each nanoparticle formulation is unique, accurate toxicity testing is needed for any proposed contrast agent in both preclinical research and potential clinical translation. To evaluate potential cytotoxicity as well as cellular internalization of the GNPs, Lewis lung carcinoma cells (LLC, American Type Culture Collection, Manassas, VA, USA) were used as the murine model of NSCLC. We performed MTT and trypan blue assays to estimate particle toxicity, and ICP-OES to quantify the gold content up-taken by the cells. Scanning transmission electron microscopy (STEM) was also used to confirm the cellular uptake of GNPs, as well as provide insight into the mechanisms of particle internalization.

#### 2.5.1. Maintenance and LLC Subculture

Murine Lewis lung carcinoma (LLC) cells were purchased from ATCC^®^ (American Type Culture Collection, Manassas, VA, USA) and cultured in either T-75 or T-175 flasks. Cells were passaged for subculturing by first aspirating the culture medium with a pipette, rinsing with 1× phosphate buffered saline (PBS, Thermo Fisher Scientific, Waltham, MA, USA, SH30256FS), aspirating off the PBS, then rinsing with 0.25% trypsin-0.53 mM EDTA solution (Thermo Fisher Scientific, Waltham, MA, USA, 25-200-056), and then they were neutralized with complete growth media consisting of Dulbecco’s modified Eagle’s medium (DMEM, ATCC^®^, Manassas, VA, USA) with 10% fetal bovine serum (FBS, USDA approved, ATCC^®^, Manassas, VA, USA), and 1% Penicillin-Streptomycin (10,000 U/mL, Thermo Fisher Scientific, Waltham, MA, USA). Cells were modified to be luciferase-expressing (LLC-Luc), as previously described [54] through use of plasmid pLenti PGK V5-LUC Neo [63] (Addgene, Cambridge, MA, USA) which was packaged in lentiviral particles and performed at the Baylor College of Medicine (BCM) vector core facility. For the luciferase-expressing cells, 1% Geneticin (Thermo Fisher Scientific, Waltham, MA, USA) was added to the media to maintain culture. Cells were maintained in a HERAcell 150i CO2 incubator (Thermo Fisher Scientific, Waltham, MA, USA) set to 37 °C and 5% humidity.

#### 2.5.2. Trypan Blue Assay

At a concentration density of 3 × 10^5^ cells/well LLC-Luc cells were seeded into 6-well plates containing 4 mL of complete media. Cells in each well were treated by adding, from a solution of ~4 mg [Au]/mL, 50 μL of either citrate or BSA-GNPs (3 μM BSA). Each treatment was performed in triplicate wells and after treatment the plates were incubated. After 24 h incubation at 37 °C and 5% humidity, the wells were washed three times with 1× PBS and detached using 0.5 mL per well of 0.25% trypsin-0.53 mM EDTA solution. Cells were then resuspended with 1 mL of complete media and 10 μL of the samples were treated with 10 μL of Trypan Blue to determine cell count and viability using a Countess™ II FL Automated Cell Counter (Invitrogen). The remaining cells were centrifuged at 100× *g* for 5 min, then the supernatant was removed, and was used for ICP-OES quantification of gold content. Cells were monitored during GNP treatment using optical microscopy (Nikon Eclipse Ts2 Microscope, Nikon Instruments Inc., Melville, NY, USA).

#### 2.5.3. Quantification of Intracellular Gold Content Using ICP-OES

ICP-OES is a common technique for the quantification of the cellular uptake of metal NPs since it offers high selectivity for elemental analysis. Measurements were performed on a Varian Agilent 720-es ICP spectrometer (Agilent, Santa Clara, CA, USA). Calibration curves for gold were obtained from a calibration standard (Au 1000 mg/mL in 10% HCl, Perkin Elmer) diluted in 1% trace metal grade nitric acid (Thermo Fisher Scientific, Waltham, MA, USA, A509) and 10% HCl (Thermo Fisher Scientific, Waltham, MA, USA, A508). Yttrium (Sigma Aldrich, St. Louis, MO, USA, Cat#01357) was used as internal standard for all ICP-OES measurements. Wavelengths of 242.794 nm and 267.594 nm were used to measure gold emission. Using the ICP-OES software (ICP Expert II, v1.1.3.b263, Sydney, Australia, Pty, Ltd 1997-2009), the gold concentration at each wavelength was calculated from the obtained calibration curve, and the measurements were averaged from both wavelengths. The reported concentrations were obtained by dividing the gold content obtained from ICP-OES by the total number of cells after 24 h of particle incubation. Then, 1 mL aqua-regia solution (nitric acid and hydrochloric acid in a molar ratio of 1:3) was added to the vial containing the pellet of the cells. The solution was placed on a hot plate (T = 60 °C) in a chemical fume hood for digestion of the cellular matrix. After complete digestion, the solution was resuspended in 10 mL of standard diluent (10% HCl, 1% Nitric Acid) and filtered using 0.6 μm filters (MilliporeSigma™ Stericup Quick Release-GP Sterile Vacuum Filtration System, Sigma, St. Louis, MO, USA).

#### 2.5.4. MTT Assay for Cytotoxicity

The MTT (3-(4,5-dimethylthiazolyl-2)-2,5-diphenyltetrazolium bromide) cell proliferation assay is used to quantify changes in the rate of cell proliferation by the reduction of tetrazolium salts and spectrophotometric measurements. LLC-Luc cells were seeded at a concentration of 4 × 10^4^ cells/well into 96-well plates and incubated overnight for adhesion. Cells were treated with different concentrations (1.5 μg[Au]/well and 5 μg[Au]/well) of particles (in a volume of 10 μL) and incubated for 24 h. After particle incubation, 10 μL of MTT Reagent (ATCC^®^, American Type Culture Collection, Manassas, VA, USA) was added to each well including control wells (consisting of either media alone, media with GNPs, or cells with media without GNPs) and incubated at 37 °C for 2 to 4 h (until a purple precipitate was visible under the microscope). Then 100 μL of Detergent Reagent (ATCC^®^, American Type Culture Collection, Manassas, VA, USA) was added to each well (including controls) and stored at room temperature in the dark for 2 to 4 h. Absorbance readings were performed at 570 nm and at 690 nm using a Synergy™ H4 Hybrid Microplate Reader (BioTek Instruments, Inc., Winooski, VT, USA).

#### 2.5.5. Scanning Transmission Electron Microscopy (STEM) to Confirm GNP Uptake

The cells treated as described in Section 2.5.1 were fixed by resuspending the pellet after centrifugation in 1 mL of 4% paraformaldehyde (Electron Microscopy Sciences, Hatfield, PA, USA). Three washes of 0.1 M PBS were performed on the samples for 10 min each. After the cells were fixed, samples were treated for 2 h at room temperature with 2% osmium tetroxide (OsO_4_) in cacodylate buffer. The samples were then washed again three times for 10 min in 0.1 M PBS followed by dehydration using a series of graded ethanol (30%, 50%, 70%, 90%) for 10 min each. The final washes used 90% acetone for 10 min and 100% acetone for 15 min repeated three times. To achieve resin embedding, the steps included the following: 2 h pre-inclusion in resin/100% acetone (1:1), overnight pre-inclusion in resin/100% acetone (2:1), 3 h pre-inclusion in 100% resin and finally, embedding in 100% resin using flat molds. To achieve complete polymerization, the samples were incubated in a 60 °C oven for 48 h and sectioned using a diamond knife to generate 100 nm ultrathin sections. These ultrathin sections were mounted on copper grids (200 mesh) (Ted Pella, Inc., Redding, CA, USA), stained with uranyl acetate and lead citrate, and imaged in a bright field setting in STEM mode and a vacuum of 15 KV with an FEI Nova NanoSEM 230 (FEI Co., Hillsboro, OR, USA).

### 2.6. In Vivo Biodistribution and Retention of GNPs

#### 2.6.1. C57BL/6 Mice and LLC Model

In vivo experiments were performed using six-week-old female C57BL/6 mice, purchased from Taconic Biosciences (Rensselaer, NY, USA). All experiments conducted on the mice were approved for study (approved code: AUP-0619-0027, 6 May 2019) by the Institutional Animal Care and Use Committee (IACUC) at the Houston Methodist Research Institute, and were performed according to the principles of the NIH Guide for the Care and Use of Laboratory Animals, the provisions of the Animal Welfare Act, PHS Animal Welfare Policy, and the policies of the Houston Methodist Research Institute. Housing and care were provided in accordance with the regulations of the Animal Welfare Act and recommendations of the Guide for the Care and Use of Laboratory Animals. Under the effects of sedation, subcutaneous injection of 2 × 10^6^ of LLC-Luc cells was performed into the right flank, when the mice weight was around 20 g. Intra-tumoral (IT) injections of either saline (control group, *n* = 4), citrate-GNPs (50 μL, 4 mg/mL (low dose) for *n* = 8 mice or 15 mg/mL (high dose) for *n* = 3 mice, pH = 6) or BSA-capped GNPs (50 μL, 4 mg/mL (low dose) for *n* = 8 mice or 15 mg/mL (high dose) for *n* = 3 mice, pH = 6, 3 μM BSA) occurred once the tumor volume reached around 100 mm^3^. All injections were performed after anesthetizing the animals with isoflurane. Injections were performed either manually or automatically using a syringe pump (KD Scientific Inc., Holliston, MA, USA) set to 0.43 μL/s. Animals were monitored daily to ensure good body condition, adequate food/water, and clean cages. CT imaging was performed pre-injection (as a baseline for the biodistribution analysis) as well as immediately post-injection, and on days 3, 6, and 9 post-injection. CT imaging was achieved using a Siemens Inveon Multi-Modality (MM) System controlled with the Inveon Acquisition Workplace (IAW), with slice thickness of 103.25 μm, in a plane resolution of 103.25 μm, tube voltage at 80 kV, tube current at 500 μA, and exposure time of 240 ms. Tumor volumes (V) in mm^3^ were calculated through daily measurements of the tumor axes using digital calipers (Figure S1) and the following Equation (3):

(3) $$V=\frac{D\;\times \;d\;\times \;d}{2}$$

where D and d represent respectively the major and the minor axis of the tumor measured in mm. The study endpoint was determined as 19 days post-tumor cell injection or tumor volume greater than 2 cm^3^, tumor interfering with normal physiological function, surgical complications, or other symptoms as outlined in the HMRI Guidelines and Policies for Determination of Humane Endpoints and Tumor Monitoring Policy, as well as the recommendation of the Comparative Medicine Program (CMP) veterinary staff.

#### 2.6.2. Determination of Au in Organs and Blood

Mouse tumors, livers, kidneys, spleens, and lungs were harvested upon euthanasia, 9 days after low dose particle injection (*n* = 8/group), and mouse tumors, livers, kidneys, spleens, lungs, hearts, brains and blood were harvested 3, 6, or 9 days after high-dose particle injection (*n* = 3/group) and were weighed and flash frozen at −80 °C. Organs and blood were then dissolved in 2 mL of fresh aqua regia, heated at 60 °C for 1 h, and left under the hood until the samples were completely dissolved. After complete digestion, the solution was resuspended in the standard diluent (10% HCl, 1% Nitric Acid) to 10 mL and filtered using 0.6 μm filters (MilliporeSigma™ Stericup Quick Release-GP Sterile Vacuum Filtration System, Sigma, St. Louis, MO, USA). Gold concentration was determined using a Varian Agilent 720-es ICP-OES spectrometer (Agilent, Santa Clara, CA, USA).

### 2.7. Statistical Analysis

GraphPad Prism 8 (version 8.3.0; GraphPad Software, Inc., San Diego, CA, USA) was used for statistical analyses. Mean ± standard error of the mean (s.e.m.) values were calculated for all results. One-way analysis of variance (ANOVA) with Tukey’s multiple comparisons test was used to assess statistical significance.

## 3. Results

### 3.1. GNP Characterization and Physicochemical (Charge, Size, Functionalization, X-ray Attenuation) Properties

**Figure 1:**
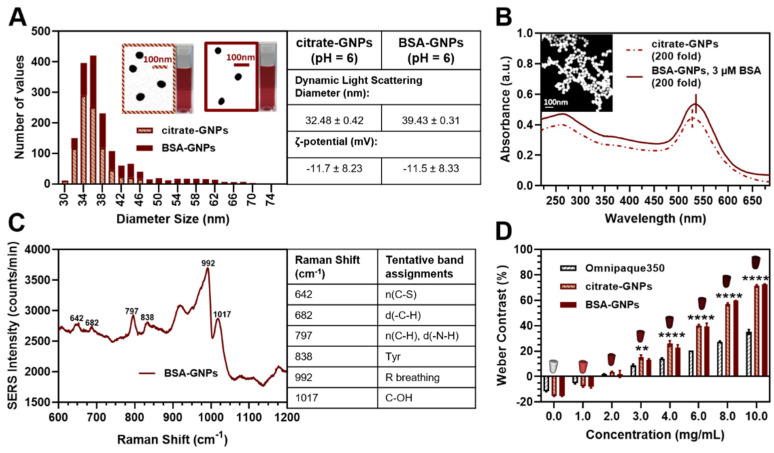
Gold nanoparticle (GNP) surface passivation and characterization. (**A**) Distribution analysis, size and charge: histograms (*n* > 700) for citrate-GNPs and GNPs functionalized with bovine serum albumin (BSA-GNPs) calculated using a MATLAB algorithm based on scanning transmission electron microscopy (STEM) images. Insert table shows dynamic light scattering (DLS) diameter and ζ-potential. (**B**) Absorbance spectrum of citrate-GNPs (dashed line) and BSA-GNPs (solid line) as well as their size and shape captured by scanning electron microscopy (SEM) (inset represents citrate-GNPs). (**C**) Surface-enhanced Raman scattering (SERS) spectrum of BSA-GNPs and table of Raman shift values and tentative band assignments. (**D**) Weber contrast calculated based on computed tomography (CT) phantom and tumor background. Citrate-GNPs and BSA-GNPs present higher X-ray attenuation properties compared to a standard contrast agent (Omnipaque350). Significant differences between GNPs (citrate-GNPs and BSA-GNPs) and standard contrast agent for concentration above 3 mg/mL (** *p* < 0.005, **** *p* < 0.0001).

Spherical GNPs were synthesized and measured with electronic microscopy to have a particle diameter of 36 ± 5 nm for the citrate-GNPs and 41 ± 8 nm for the BSA-coated GNPs (mean ± SD). Since the optical properties of spherical GNPs are dependent on particle diameter, we chose this particle size as it produced a strong SPR peak. While smaller particles might diffuse more easily and faster, the SPR band for GNPs with sizes smaller than 10 nm is largely damped [16]. Finally, it should be noted that the CT contrast properties are not dependent on particle size [64]. Histograms of both particle types are shown in Figure 1A. Unfunctionalized and BSA-functionalized particles appeared similar in color, were spherical and well-rounded, and had low polydispersity (insets of Figure 1A). The hydrodynamic diameters of the GNPs as measured by DLS were within the error of the core diameters estimated from STEM. GNP surface charges were found to be negative, as expected. Both sample types displayed similar optical absorption spectra (Figure 1B) in the UV-VIS. The 4 nm red shift of the SPR peak (from 530 nm to 534 nm) and representative SERS spectrum of BSA with gold nanoparticles (Figure 1C) confirm protein surface passivation. The assigned band positions are in accordance with previous studies [65]. CT phantom imaging was performed to demonstrate the high X-ray attenuation properties of the particles. As shown in Figure 1D, the change in the attenuation levels expressed by the percentage variation in the Weber contrast compared to the tumor background linearly correlates with GNP concentration. Both citrate-GNPs and BSA-GNPs present significantly greater attenuation values compared to a standard contrast agent (Omnipaque350) for a concentration above 3 mg [Au]/mL (** *p* < 0.005, **** *p* < 0.0001). No significance was highlighted between the citrate-GNPs and BSA-GNPs.

### 3.2. BSA Adsorption Models on GNPs

**Figure 2:**
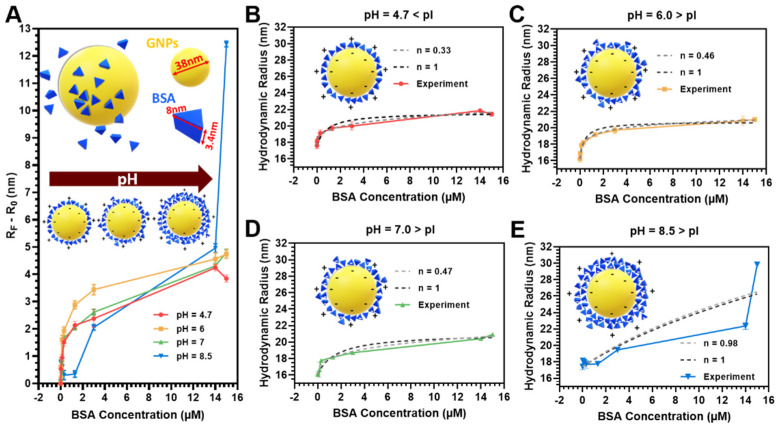
Adsorption isotherms (T = 25 °C) showing hydrodynamic radii experimentally determined with dynamic light scattering as a function of BSA concentration and pH. (**A**) Adsorption of BSA on the GNP surface at different pH values above and below the isoelectric point (pI) of BSA (pI = 5, Ge et al. [67]). The y-axis in (**A**) was calculated by subtracting the hydrodynamic radii of the citrate-GNPs (R0) at their respective pH from the hydrodynamic radius of each adsorption point experimentally determined (RF). Data are fit using the Langmuir model following the approach of Dominguez-Medina et al. [61] and the results are reported for (**B**) pH = 4.7, (**C**) pH = 6.0, (**D**) pH = 7.0, and (**E**) pH = 8.5. Isotherms with returned best fit Hill coefficient where n is variable (gray dashed line) or non-cooperative binding model where *n* = 1 (dashed black line). The adsorption isotherms for 35 nm GNPs at pH 4.7, 6.0, and 7.0 follow an anti-cooperative binding model, while at pH 8.5 they follow a cooperative binding model.

After the citrate-reduction synthesis, GNPs can have acidic or slightly acidic pH (in the range of 3–6) depending on relative concentrations and particle sizes [66]. Without any pH modifications, our particle solution’s pH is 3.5. Figure 2 shows the adsorption isotherms obtained by fitting the hydrodynamic radii, experimentally determined with DLS as a function of BSA concentration and pH, with the model described in Section 2.4.2. BSA adsorbs saturates according to the predicted model. The dotted lines (black and gray) in Figure 2B–E respectively represent the Langmuir model for adsorption (with Hill’s coefficient *n* = 1) and the modified Langmuir model (where *n* < 1) for anti-cooperative binding, which indicates strong repulsive forces between free and bound BSA molecules that increase in number as more binding sites on the surface become occupied. The adsorption isotherm for 35 nm GNPs at pH 4.7, 6.0, and 7.0 follows an anti-cooperative binding model, while at pH = 8.5, it follows a cooperative binding model. Adsorption beyond a monolayer is predicted to be negligible for GNPs at pH 4.7, 6.0, and 7.0.

**Figure 3:**
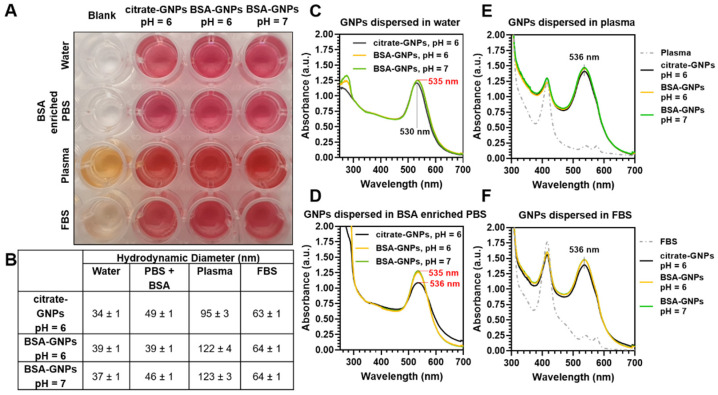
Optical photo, hydrodynamic diameter, and UV-Vis spectra of gold nanoparticles (GNPs) as a function of surface functionalization and immobilization media. (**A**) Photographic image showing wells containing citrate-GNPs (pH = 6) and BSA-GNPs (3 μM, pH = 6 or pH = 7) dispersed in water, 600 μM BSA-enriched phosphate buffered saline (PBS), plasma, and fetal bovine serum (FBS). (**B**) Dynamic light scattering (DLS) measurements show changes in hydrodynamic diameter as a function of surface functionalization and dispersion media at these pH values. (**C**–**F**) UV-Vis extinction spectra of citrate-GNPs and BSA-GNPs dispersed in (**C**) water, (**D**) 600 μM BSA enriched PBS, (**E**) plasma, and (**F**) FBS.

We also monitored the citrate-GNP and BSA-GNP particles (3 μM) visually and spectroscopically, as well as changes in the hydrodynamic diameter when resuspended in either water, a solution of 600 μM BSA-enriched PBS (which corresponds to the average physiological level of proteins), plasma, or FBS (Figure 3) to mimic a simulated physiological environment. We observed (Figure 3A) no macro-aggregation or flocculation phenomena, as well as no visible change in color, when dispersing the particles in a source of protein as a function of surface functionalization at these pH values. We also found that the hydrodynamic diameter of the particles (Figure 3B) increased when the particles were dispersed in media containing an external source of protein, whereby the increasing trend in size went from water to BSA-enriched PBS, to FBS, and finally to plasma. Interestingly, in the plasma, the sizes were larger for the BSA-GNPs than the citrate-GNPs, indicating that proteins from the plasma were likely adding layers to the corona rather than displacing the BSA. The DLS data are complemented by the UV-Vis spectra (Figure 3C–F) of the particles in various media. There is an observed SPR shift from 530 to 535 nm when the particles are coated with BSA in water (Figure 3C), and this shift remains apparent in all three protein-rich media (Figure 3E,F) even for the citrate-GNPs, indicating that they become passivated with protein from the media under these conditions. We decided to maintain GNPs at pH = 6.0 for the in vivo experiments, because they are stable in a simulated physiological environment and their pH is closer to the extracellular pH of the tumor environment, which is slightly acidic (6.0–7.4) because of the extra secretion of lactic acid and CO_2_ by the tumor cells [68].

### 3.3. In Vitro Uptake of GNPs

**Figure 4:**
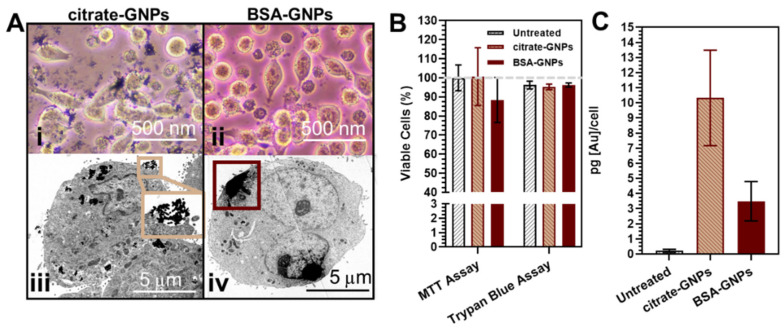
Evaluation of particle uptake and viability with LLC-Luc cells. (**A**) Optical microscopy (i,ii) and STEM (iii,iv) images of cells treated and incubated for 24 h. (**B**) 3-(4,5-dimethylthiazol-2-yl)-2,5-diphenyltetrazolium bromide (MTT) and Trypan Blue assays for cells treated and incubated for 24 h. No significant differences between groups. (**C**) Quantification of GNPs internalized in LLC-Luc by ICP-OES after 24 h. In (**B**) and (**C**) cells were treated and incubated in triplicate wells and data are plotted as the mean with s.e.m. Dashed line represents 100% cell viability.

In vitro assessments with optical microscopy of the LLC-Luc cells incubated with citrate-GNPs and BSA-GNPs after 24 h showed aggregation of the citrate-GNPs (Figure 4Ai), while the BSA-GNPs remained stable and micro-clusters of the particles were not visible (Figure 4Aii). The intracellular content of gold after incubation of the LLC-Luc cells with citrate-GNPs and BSA-GNPs for 24 h was clearly visible with electronic microscopy (Figure 4Aiii–iv). The inset of Figure 4Aiii shows particle internalization by macropinocytosis, in which the particles are taken into an endocytic vesicle in a nonspecific bulk fluid uptake. We noticed that the BSA-GNPs are internalized in larger vesicles (1–2 μm), while in the case of citrate-GNPs, macropinocytosis is accompanied by the presence of particles uptaken by endosomes in different stages of maturation (early endosome, late endosome and lysosome). This is not surprising as GNPs have been found to undergo both endocytosis and exocytosis patterns in cells [69]. Analysis of MTT and Trypan blue assays after 24 h of particle incubation shows that a high concentration of functionalized and unfunctionalized nanoparticles does not impact cytotoxicity (Figure 4B). The lack of any noticeable toxicity from citrate-GNPs and BSA-GNPs, or cell proliferation suppression compared to untreated cells, provides evidence for their safe application in vivo. As described in Section 2.5.3, ICP-OES was used to quantify the Au mass in the LLC-Luc cells. Figure 4C shows Au concentration per cell. No statistical significance is highlighted between groups, although the citrate-GNP content is higher than the BSA-GNP content in LLC-Luc cells. This result can be partially explained by the saturated protein corona formed in the case of BSA-GNPs, which decreases the uptake efficacy of GNPs by cells.

### 3.4. In Vivo Biodistribution and Retention of GNPs

The biodistribution and retention of citrate-GNPs and BSA-GNPs were assessed using in vivo CT imaging. Mice were pre-scanned, injected intratumorally with GNPs as described in Section 2.6.1, and imaged at various time points with a micro-CT system.

#### 3.4.1. Manual Intratumoral Injection of Low Dose GNPs

**Figure 5:**
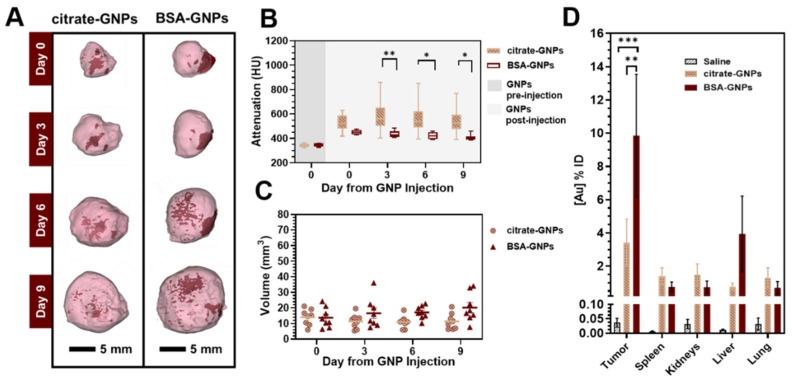
Micro-CT and ICP-OES results both confirm that particles remain in the tumor 9 days post-injection. (**A**) Representative 3D volume renderings of micro-CT images of concentrated GNPs manually intratumorally injected into solid LLC-Luc tumors grown on the right flanks of C57BL/6 mice (9 days follow up). Images are rendered at a window level of 1090 HU with 930 HU window width. With this color look-up table, solid tumors are shown in pink (40% transparency) and contrast arising from the injected GNPs is shown in dark red (GNP clusters). Images are displayed with a voxel size of 100 μm. (**B**) Mean attenuation values in Hounsfield units (HU) calculated within the GNP cluster volume over time using CT follow-up images. Significant difference in BSA-GNPs vs. saline group and citrate-GNPs over time (* *p* < 0.05, ** *p* < 0.005 Tukey’s multiple comparisons test). (**C**) GNP cluster volume over time calculated using CT follow-up images. (**D**) Biodistribution of citrate-GNPs and BSA-GNPs in digested organs 9 days post-injection (*n* = 8 mice per group) using ICP-OES. Saline injections were performed on 4 mice as a negative control group (black bars). A two-way ANOVA test was done to compare the interactions between each group. Significant difference of BSA-GNPs vs. saline group and citrate-GNPs (** *p* < 0.005, *** *p* < 0.0005 Tukey’s multiple comparisons test).

Representative CT images acquired from follow-up scans after manual low-dose (50 μL of 4 mg/mL) GNP injection are shown in Figure 5A and indicate that the injected nanoparticles produce strong CT contrast. At 9 days post-injection, we were still able to locate particles in the tumor volume. We quantified the intratumoral contrast at different time points (Figure 5B) as well as the volume of the visible particles diffused in the tumor area (Figure 5C). Mean attenuation values were calculated in Hounsfield units (HU) within the GNP volume over time. Sustained strong CT contrast highlighted a significant difference between the BSA-GNPs and both the control group (saline) and citrate-GNPs (* *p* < 0.05, ** *p* < 0.005). This result is consistent with the increase in BSA-GNP volume over time in Figure 5C: the more the BSA-GNPs diffuse throughout the tissues, the less they attenuate the X-rays. Citrate-GNP volume is constant over time, confirming the preliminary results we previously obtained [55]: citrate-GNPs do not diffuse over time intratumorally, but instead form a single cluster of particles. Overall, a heterogenic intratumoral distribution pattern was found for both citrate-GNPs and BSA-GNPs. However, we cannot exclude that particles may be forcefully spread out by the tissue as the tumor grows. The BSA-GNPs particles accumulated mostly in the tumor periphery, even though smaller depositions were found throughout the whole tumor region. Elemental analysis performed with ICP-OES (Figure 5D) also confirms the presence of the particles in the tumor 9 days post-injection. Significant differences are highlighted between the saline and BSA-GNP groups (*** *p* < 0.0005, Tukey’s multiple comparisons test) and the citrate-GNP and BSA-GNP groups (** *p* < 0.005, Tukey’s multiple comparisons test). This outcome validates the hypothesis that the protein corona of GNPs influences the intratumoral distribution and retention of nanoparticles. We also performed elemental analysis for the spleen, kidneys, liver, and lungs of the mice 9 days post-GNP injection. Although both particles types were administered through intratumoral injection, we surprisingly saw evidence of gold content in these other organs; however, no statistical significance was determined.

#### 3.4.2. Automatic Syringe Pump Intratumoral Injection of High Dose GNPs

**Figure 6:**
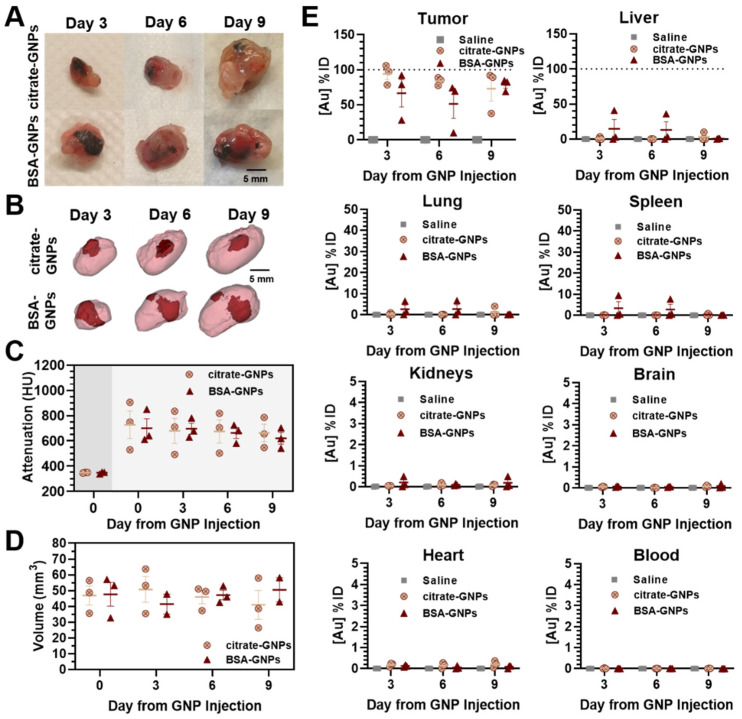
Micro-CT and ICP-OES demonstrate particle distribution at different time points after high-dose intratumoral injection using an injection pump. (**A**) Ex vivo LLC solid tumors at different sacrifice time points. (**B**) three-dimensional renderings of the intratumoral biodistribution of citrate-GNPs and BSA-GNPs from micro-CT images. Residual intratumoral GNPs are rendered in dark red, tumor tissue is rendered in pink (40% transparency). (**C**) Mean attenuation values in Hounsfield units (HU) calculated within the GNP cluster volume over time using CT follow-up images (GNP pre-injection: dark gray area; GNP post-injection: dark gray area). (**D**) GNP cluster volume over time calculated using CT follow-up images. (**E**) Biodistribution of citrate-GNPs and BSA-GNPs in digested tumors, organs (liver, lung, spleen, kidneys, brain, heart), and blood over time (*n* = 3 mice per group) using ICP-OES. Saline injections were performed on 3 mice as a negative control group. All data in the figure are reported as mean ± standard error of the mean (s.e.m). Dashed lines represent 100% of the injected dose.

The ex vivo photographs of tumors taken after sacrificing the mice on days 3, 6, and 9 post-injection (Figure 6A), and representative CT images (Figure 6B) with corresponding mean attenuation values (Figure 6C) acquired from follow-up scans after automatic high-dose (50 μL of 15 mg/mL) GNP injection, allow for the comparison of intratumoral distribution. Data reported here are consistent with the results shown in Figure 5, and the higher-dose citrate-GNPs also appear more as a single central cluster of particles, while the BSA-GNPs are more diffuse around the tumor edge. Figure 6D shows the volume of GNPs quantified intratumorally for both treatment groups over time. When compared with Figure 5C, where we observe less than 25 mm^3^ in volume (equating to less than 50% of the injected volume in the tumor), under these conditions the volume is around 50 mm^3^ (equating to close to 100% of the injected dose). Additionally, the gold quantified in the solid tumors from ICP-OES analyses was close to 100% for both treatment groups. We did notice a drop in the percent of gold accumulated in the tumors of two BSA-GNP-injected mice—one from day 3 and one from the day 6 group. In most of the other organs and blood analyzed, the quantity of gold measured was negligible except for in the liver, lung, and spleen of these same two mice. Overall, we attribute the greater percent of gold quantification in the tumors through both CT and ICP-OES (shown in Figure 6) to the use of the injection pump and a more concentrated sample.

## 4. Discussion

In this work, we demonstrated the fabrication and characterization of citrate-stabilized and BSA-surface-passivated GNPs, assessed their cellular uptake and lack of cytotoxicity in vitro, and evaluated their biodistribution and retention in an in vivo murine model of NSCLC. Recent studies have demonstrated the significant effects of albumin on the physicochemical properties of nanoparticles, which inhibits plasma protein adsorption and decreases blood clearance time [70,71]. However, there still remains a lack of knowledge regarding how the surface chemistry of GNPs can influence particle distribution within a tumor microenvironment. Evaluating and providing better understanding of the effects of surface passivation may help advance their clinical translation as theranostic tools.

Several works have studied protein corona formation after the intravenous injection of particles [13,72]. The advantage of the direct administration of nanomaterials into the bloodstream is related to their rapid distribution throughout the vasculature. However, this feature can also result in a rapid clearance by organs, such as the kidneys and liver or the reticuloendothelial system. In this study, we overcome this limitation through intratumoral injection. This approach enables the administration of highly concentrated nanoformulations. With this strategy, we were able to demonstrate that the particles are retained intratumorally for 9 days, without significant accumulation in other organs.

Although intratumoral injections enable direct delivery of the nanomaterial into the interstitium of cancerous tissue, the interstitial tumoral pressure is higher than in healthy tissue. This elevated interstitial fluid pressure gradient generally pushes the injected formulation out of the tumor and produces higher leakage of the drug into the surrounding tissue. Identifying the intra-tumoral distribution of nanoparticles is clinically relevant because it can help determine the success of nanomedicine-based therapy and has often been considered a physical mechanism of drug resistance.

It is now known that both chemical and biological components play roles in the radiosensitization process in addition to physical processes [73]; therefore, it is expected that the observed different distribution patterns dependent on the particle functionalization would affect radiosensitization. These effects may be due to aspects such as the quantity and distance of nearby particles, and the percent uptake and location of the particles inside or near various cell types undergoing different phases of replication. For example, it is generally understood that cells that are dividing quickly and are highly active metabolically are more radiosensitive. Therefore, protein surface modification that results in higher particle distribution in high-turnover cells would likely further enhance radiosensitization effects, which may prove useful in future clinical application.

In vivo results from this study show that the intratumoral biodistribution of GNPs is dependent on surface passivation and can result in significant heterogeneity throughout the tumor microenvironment. Predominately, the perfusion of BSA-GNPs occurs throughout the tumor periphery with reduced deposition covering the entire tumor volume. While some of this could be attributed to off-centered injection or the fact that tumor growth will further spread out the particle distribution, we attribute the majority of this response to the abnormal and heterogeneous vascular structure of the LLC tumor, suggesting perfusion rather than the permeability of the GNPs as the limiting factor for tumor accumulation. Despite perivascular accumulation, we demonstrate that BSA surface passivation can affect the intratumoral distribution and retention of GNPs.

## 5. Conclusions

In summary, we investigated whether protein surface adsorption can influence GNP biodistribution in an NSCLC animal model by applying high-resolution preclinical CT imaging. By controlling protein absorption on the GNP surface, we obtained a significant difference in the intratumoral distribution and retention of the particles, as demonstrated through quantitative CT and ICP-OES analysis. Moreover, our investigations revealed that the surface passivation of GNPs controls the mechanism of cellular uptake in vitro. Further evaluation will expand our knowledge of how to better control the surface passivation of GNPs and prove useful for the clinical translation of nanoparticle-based therapies.

## Data Availability

The data that support the findings of this study are available from the corresponding author upon reasonable request.

## Supplementary Materials

The following are available online at https://www.mdpi.com/1999-4923/13/2/216/s1, Figure S1. Mice weight and tumor measurements. (A) Body weight of female black mice (*n* = 8/group). (B) Tumor volume measured using a caliper (*n* = 8/group). (C) Comparison between tumor volumes measured manually by external caliper or quantified from micro-CT analysis (*n* = 16 mice for each measurement modality). No significant differences highlighted. Each data point in A–C represents the mean ± standard error of the mean (s.e.m.). (D) Data are shown in boxplots for Day 10. (E) Bland Altman comparison between tumor volumes measured manually by external caliper or quantified from micro-CT analysis. A bias of 15 mm^3^ is detected.

## Abbreviations

ANOVA	analysis of variance
BSA	Bovine Serum Albumin
CT	Computed Tomography
DLS	Dynamic light scattering
EPR	enhanced permeability and retention effect
FBS	fetal bovine serum
GNP	gold nanoparticle
HU	Hounsfield unit
IACUC	Institutional Animal Care and Use Committee
IAW	Inveon Acquisition Workplace
ICP-OES	Inductively Coupled Plasma Optical Emission Spectrometry
IT	intra-tumoral
LLC	Lewis Lung Carcinoma
LLC-Luc	luciferase expressing Lewis Lung Carcinoma
MM	Multi-Modality
MTT	(3-(4,5-dimethylthiazolyl-2)-2,5-diphenyltetrazolium bromide)
NSCLC	Non-Small Cell Lung Cancer
OsO_4_	osmium tetroxide
PBS	phosphate buffered saline
PDI	polydispersity index
pI	Isoelectric Point
ROI	region of interest
s.e.m.	standard error of the mean
SERS	Surface-enhanced Raman scattering
SPR	surface plasmon resonance
STEM	Scanning transmission electron microscopy

## References

[1] Sumer B., Gao J. (2008). Theranostic Nanomedicine for Cancer. Nanomedicine.

[2] Chua C.Y.X., Ho J., Demaria S., Ferrari M., Grattoni A. (2020). Emerging Technologies for Local Cancer Treatment. Adv. Therap..

[3] Singh D., Dilnawaz F., Sahoo S.K. (2020). Challenges of Moving Theranostic Nanomedicine into the Clinic. Nanomedicine.

[4] Auría-Soro C., Nesma T., Juanes-Velasco P., Landeira-Viñuela A., Fidalgo-Gomez H., Acebes-Fernandez V., Gongora R., Almendral Parra M.J., Manzano-Roman R., Fuentes M. (2019). Interactions of Nanoparticles and Biosystems: Microenvironment of Nanoparticles and Biomolecules in Nanomedicine. Nanomaterials.

[5] Vasti C., Bedoya D.A., Rojas R., Giacomelli C.E. (2016). Effect of the Protein Corona on the Colloidal Stability and Reactivity of LDH-Based Nanocarriers. J. Mater. Chem. B.

[6] Gebauer J.S., Malissek M., Simon S., Knauer S.K., Maskos M., Stauber R.H., Peukert W., Treuel L. (2012). Impact of the Nanoparticle–Protein Corona on Colloidal Stability and Protein Structure. Langmuir.

[7] Strojan K., Leonardi A., Bregar V.B., Križaj I., Svete J., Pavlin M. (2017). Dispersion of Nanoparticles in Different Media Importantly Determines the Composition of Their Protein Corona. PLoS ONE.

[8] Böhmert L., Voß L., Stock V., Braeuning A., Lampen A., Sieg H. (2020). Isolation Methods for Particle Protein Corona Complexes from Protein-Rich Matrices. Nanoscale Adv..

[9] Barbir R., Goessler W., Ćurlin M., Micek V., Milić M., Vuković B., Milić M., Ljubojević M., Jurašin D.D., Vrček I.V. (2019). Protein Corona Modulates Distribution and Toxicological Effects of Silver Nanoparticles In Vivo. Part. Part. Syst. Charact..

[10] Bertrand N., Grenier P., Mahmoudi M., Lima E.M., Appel E.A., Dormont F., Lim J.-M., Karnik R., Langer R., Farokhzad O.C. (2017). Mechanistic Understanding of in Vivo Protein Corona Formation on Polymeric Nanoparticles and Impact on Pharmacokinetics. Nat. Commun..

[11] Giri K., Kuschnerus I., Ruan J., Garcia-Bennett A.E. (2020). Influence of a Protein Corona on the Oral Pharmacokinetics of Testosterone Released from Mesoporous Silica. Adv. Ther..

[12] Tavakoli S., Kari O.K., Turunen T., Lajunen T., Schmitt M., Lehtinen J., Tasaka F., Parkkila P., Ndika J., Viitala T. (2020). Diffusion and Protein Corona Formation of Lipid-Based Nanoparticles in the Vitreous Humor: Profiling and Pharmacokinetic Considerations. Mol. Pharm..

[13] Corbo C., Molinaro R., Parodi A., Toledano Furman N.E., Salvatore F., Tasciotti E. (2016). The Impact of Nanoparticle Protein Corona on Cytotoxicity, Immunotoxicity and Target Drug Delivery. Nanomedicine.

[14] Mohammad-Beigi H., Hayashi Y., Zeuthen C.M., Eskandari H., Scavenius C., Juul-Madsen K., Vorup-Jensen T., Enghild J.J., Sutherland D.S. (2020). Mapping and Identification of Soft Corona Proteins at Nanoparticles and Their Impact on Cellular Association. Nat. Commun..

[15] Westmeier D., Chen C., Stauber R.H., Docter D. (2015). The Bio-Corona and Its Impact on Nanomaterial Toxicity. Eur. J.Nanomed..

[16] Huang X., El-Sayed M.A. (2010). Gold Nanoparticles: Optical Properties and Implementations in Cancer Diagnosis and Photothermal Therapy. J. Adv. Res..

[17] Huang X., Jain P.K., El-Sayed I.H., El-Sayed M.A. (2007). Gold Nanoparticles: Interesting Optical Properties and Recent Applications in Cancer Diagnostics and Therapy. Nanomedicine.

[18] Stetsenko M.O., Rudenko S.P., Maksimenko L.S., Serdega B.K., Pluchery O., Snegir S.V. (2017). Optical Properties of Gold Nanoparticle Assemblies on a Glass Surface. Nanoscale Res. Lett..

[19] Shukla R., Bansal V., Chaudhary M., Basu A., Bhonde R.R., Sastry M. (2005). Biocompatibility of Gold Nanoparticles and Their Endocytotic Fate inside the Cellular Compartment: A Microscopic Overview. Langmuir.

[20] Li X., Wang L., Fan Y., Feng Q., Cui F. Biocompatibility and Toxicity of Nanoparticles and Nanotubes. https://www.hindawi.com/journals/jnm/2012/548389/.

[21] Kumar S., Jha I., Mogha N.K., Venkatesu P. (2020). Biocompatibility of Surface-Modified Gold Nanoparticles towards Red Blood Cells and Haemoglobin. Appl. Surf. Sci..

[22] Guo J., Rahme K., He Y., Li L.-L., Holmes J.D., O’Driscoll C.M. (2017). Gold Nanoparticles Enlighten the Future of Cancer Theranostics. Int. J. Nanomed..

[23] Anderson L.J.E., Payne C.M., Zhen Y.-R., Nordlander P., Hafner J.H. (2011). A Tunable Plasmon Resonance in Gold Nanobelts. Nano Lett..

[24] Lal S., Hafner J.H., Halas N.J., Link S., Nordlander P. (2012). Noble Metal Nanowires: From Plasmon Waveguides to Passive and Active Devices. Acc. Chem. Res..

[25] Nehl C.L., Liao H., Hafner J.H. (2006). Optical Properties of Star-Shaped Gold Nanoparticles. Nano Lett..

[26] Brinson B.E., Lassiter J.B., Levin C.S., Bardhan R., Mirin N., Halas N.J. (2008). Nanoshells Made Easy: Improving Au Layer Growth on Nanoparticle Surfaces. Langmuir.

[27] Kelly A.T., Filgueira C.S., Schipper D.E., Halas N.J., Whitmire K.H. (2017). Gold Coated Iron Phosphide Core–Shell Structures. RSC Adv..

[28] Levin C.S., Hofmann C., Ali T.A., Kelly A.T., Morosan E., Nordlander P., Whitmire K.H., Halas N.J. (2009). Magnetic-Plasmonic Core-Shell Nanoparticles. ACS Nano.

[29] Choi M.-R., Stanton-Maxey K.J., Stanley J.K., Levin C.S., Bardhan R., Akin D., Badve S., Sturgis J., Robinson J.P., Bashir R. (2007). A Cellular Trojan Horse for Delivery of Therapeutic Nanoparticles into Tumors. Nano Lett..

[30] Rastinehad A.R., Anastos H., Wajswol E., Winoker J.S., Sfakianos J.P., Doppalapudi S.K., Carrick M.R., Knauer C.J., Taouli B., Lewis S.C. (2019). Gold Nanoshell-Localized Photothermal Ablation of Prostate Tumors in a Clinical Pilot Device Study. Proc. Natl. Acad. Sci. USA.

[31] Bros M., Nuhn L., Simon J., Moll L., Mailänder V., Landfester K., Grabbe S. (2018). The Protein Corona as a Confounding Variable of Nanoparticle-Mediated Targeted Vaccine Delivery. Front. Immunol..

[32] Brun E., Sicard-Roselli C. (2014). Could Nanoparticle Corona Characterization Help for Biological Consequence Prediction?. Cancer Nanotechnol..

[33] Ma Y., Hong J., Ding Y. (2020). Biological Behavior Regulation of Gold Nanoparticles via the Protein Corona. Adv. Healthc. Mater..

[34] Chen D., Ganesh S., Wang W., Amiji M. (2020). Protein Corona-Enabled Systemic Delivery and Targeting of Nanoparticles. AAPS J..

[35] Piella J., Bastús N.G., Puntes V. (2017). Size-Dependent Protein–Nanoparticle Interactions in Citrate-Stabilized Gold Nanoparticles: The Emergence of the Protein Corona. Bioconj. Chem..

[36] Li B., Lane L.A. (2019). Probing the Biological Obstacles of Nanomedicine with Gold Nanoparticles. Wiley Interdiscip. Rev. Nanomed. Nanobiotechnol..

[37] Mosquera J., García I., Henriksen-Lacey M., Martínez-Calvo M., Dhanjani M., Mascareñas J.L., Liz-Marzán L.M. (2020). Reversible Control of Protein Corona Formation on Gold Nanoparticles Using Host–Guest Interactions. ACS Nano.

[38] Elechalawar C.K., Hossen M.N., McNally L., Bhattacharya R., Mukherjee P. (2020). Analysing the Nanoparticle-Protein Corona for Potential Molecular Target Identification. J. Control. Release.

[39] Park S.J. (2020). Protein–Nanoparticle Interaction: Corona Formation and Conformational Changes in Proteins on Nanoparticles. Int. J. Nanomed..

[40] Nierenberg D., Khaled A.R., Flores O. (2018). Formation of a Protein Corona Influences the Biological Identity of Nanomaterials. Rep. Pract. Oncol. Radiother..

[41] Varga Z., Fehér B., Kitka D., Wacha A., Bóta A., Berényi S., Pipich V., Fraikin J.-L. (2020). Size Measurement of Extracellular Vesicles and Synthetic Liposomes: The Impact of the Hydration Shell and the Protein Corona. Colloids Surf. B Biointerfaces.

[42] Digiacomo L., Palchetti S., Giulimondi F., Pozzi D., Chiozzi R.Z., Capriotti A.L., Laganà A., Caracciolo G. (2019). The Biomolecular Corona of Gold Nanoparticles in a Controlled Microfluidic Environment. Lab. Chip..

[43] Cheng X., Tian X., Wu A., Li J., Tian J., Chong Y., Chai Z., Zhao Y., Chen C., Ge C. (2015). Protein Corona Influences Cellular Uptake of Gold Nanoparticles by Phagocytic and Nonphagocytic Cells in a Size-Dependent Manner. ACS Appl. Mater. Interfaces.

[44] Blanco E., Shen H., Ferrari M. (2015). Principles of Nanoparticle Design for Overcoming Biological Barriers to Drug Delivery. Nat. Biotechnol..

[45] Chandran P., Riviere J.E., Monteiro-Riviere N.A. (2017). Surface Chemistry of Gold Nanoparticles Determines the Biocorona Composition Impacting Cellular Uptake, Toxicity and Gene Expression Profiles in Human Endothelial Cells. Nanotoxicology.

[46] Han M., Zhu L., Mo J., Wei W., Yuan B., Zhao J., Cao C. (2020). Protein Corona and Immune Responses of Borophene: A Comparison of Nanosheet–Plasma Interface with Graphene and Phosphorene. ACS Appl. Bio Mater..

[47] Neagu M., Piperigkou Z., Karamanou K., Engin A.B., Docea A.O., Constantin C., Negrei C., Nikitovic D., Tsatsakis A. (2017). Protein Bio-Corona: Critical Issue in Immune Nanotoxicology. Arch. Toxicol..

[48] Oh J.Y., Kim H.S., Palanikumar L., Go E.M., Jana B., Park S.A., Kim H.Y., Kim K., Seo J.K., Kwak S.K. (2018). Cloaking Nanoparticles with Protein Corona Shield for Targeted Drug Delivery. Nat. Commun..

[49] Wang B., He X., Zhang Z., Zhao Y., Feng W. (2013). Metabolism of Nanomaterials in Vivo: Blood Circulation and Organ Clearance. Acc. Chem. Res..

[50] Gullino P.M., Clark S.H., Grantham F.H. (1964). The Interstitial Fluid of Solid Tumors. Cancer Res..

[51] Chen Z., Fillmore C.M., Hammerman P.S., Kim C.F., Wong K.-K. (2014). Non-Small-Cell Lung Cancers: A Heterogeneous Set of Diseases. Nat. Rev. Cancer.

[52] Van Lamsweerde A.L., Henry N., Vaes G. (1983). Metastatic Heterogeneity of Cells from Lewis Lung Carcinoma. Cancer Res..

[53] Dagogo-Jack I., Shaw A.T. (2018). Tumour Heterogeneity and Resistance to Cancer Therapies. Nat. Rev. Clin. Oncol..

[54] Pandey A., Vighetto V., Di Marzio N., Ferraro F., Hirsch M., Ferrante N., Mitra S., Grattoni A., Filgueira C.S. (2020). Gold Nanoparticles Radio-Sensitize and Reduce Cell Survival in Lewis Lung Carcinoma. Nanomaterials.

[55] Terracciano R., Sprouse M.L., Wang D., Ricchetti S., Hirsch M., Ferrante N., Butler E.B., Demarchi D., Grattoni A., Filgueira C.S. Intratumoral Gold Nanoparticle-Enhanced CT Imaging: An in Vivo Investigation of Biodistribution and Retention. Proceedings of the IEEE 20th International Conference on Nanotechnology.

[56] Hughes H.J., Demers S.M.E., Zhang A., Hafner J.H. (2020). The Orientation of a Membrane Probe from Structural Analysis by Enhanced Raman Scattering. Biochim. Biophys. Acta Biomembr..

[57] Fedorov A., Beichel R., Kalpathy-Cramer J., Finet J., Fillion-Robin J.-C., Pujol S., Bauer C., Jennings D., Fennessy F., Sonka M. (2012). 3D Slicer as an Image Computing Platform for the Quantitative Imaging Network. Magn. Reson. Imaging.

[58] Baimanov D., Cai R., Chen C. (2019). Understanding the Chemical Nature of Nanoparticle–Protein Interactions. Bioconjugate Chem..

[59] Sotnikov D.V., Berlina A.N., Ivanov V.S., Zherdev A.V., Dzantiev B.B. (2019). Adsorption of Proteins on Gold Nanoparticles: One or More Layers?. Colloids Surf. B Biointerfaces.

[60] Jommanee N., Chanthad C., Manokruang K. (2018). Preparation of Injectable Hydrogels from Temperature and PH Responsive Grafted Chitosan with Tuned Gelation Temperature Suitable for Tumor Acidic Environment. Carbohydr. Polym..

[61] Dominguez-Medina S., McDonough S., Swanglap P., Landes C.F., Link S. (2012). In Situ Measurement of Bovine Serum Albumin Interaction with Gold Nanospheres. Langmuir.

[62] Röcker C., Pötzl M., Zhang F., Parak W.J., Nienhaus G.U. (2009). A Quantitative Fluorescence Study of Protein Monolayer Formation on Colloidal Nanoparticles. Nat. Nanotech..

[63] Campeau E., Ruhl V.E., Rodier F., Smith C.L., Rahmberg B.L., Fuss J.O., Campisi J., Yaswen P., Cooper P.K., Kaufman P.D. (2009). A Versatile Viral System for Expression and Depletion of Proteins in Mammalian Cells. PLoS ONE.

[64] Dong Y.C., Hajfathalian M., Maidment P.S.N., Hsu J.C., Naha P.C., Si-Mohamed S., Breuilly M., Kim J., Chhour P., Douek P. (2019). Effect of Gold Nanoparticle Size on Their Properties as Contrast Agents for Computed Tomography. Sci. Rep..

[65] Szekeres G.P., Kneipp J. (2019). SERS Probing of Proteins in Gold Nanoparticle Agglomerates. Front. Chem..

[66] Tyagi H., Kushwaha A., Kumar A., Aslam M. (2016). A Facile PH Controlled Citrate-Based Reduction Method for Gold Nanoparticle Synthesis at Room Temperature. Nanoscale Res. Lett..

[67] Ge S., Kojio K., Takahara A., Kajiyama T. (1998). Bovine Serum Albumin Adsorption onto Immobilized Organotrichlorosilane Surface: Influence of the Phase Separation on Protein Adsorption Patterns. J. Biomater. Sci. Polym. Ed..

[68] Sutoo S., Maeda T., Suzuki A., Kato Y. (2020). Adaptation to Chronic Acidic Extracellular PH Elicits a Sustained Increase in Lung Cancer Cell Invasion and Metastasis. Clin. Exp. Metastasis.

[69] Oh N., Park J.-H. (2014). Endocytosis and Exocytosis of Nanoparticles in Mammalian Cells. Int. J. Nanomed..

[70] Mocan L., Matea C., Tabaran F.A., Mosteanu O., Pop T., Puia C., Agoston-Coldea L., Zaharie G., Mocan T., Buzoianu A.D. (2017). Selective Ex Vivo Photothermal Nano-Therapy of Solid Liver Tumors Mediated by Albumin Conjugated Gold Nanoparticles. Biomaterials.

[71] Charbgoo F., Nejabat M., Abnous K., Soltani F., Taghdisi S.M., Alibolandi M., Thomas Shier W., Steele T.W.J., Ramezani M. (2018). Gold Nanoparticle Should Understand Protein Corona for Being a Clinical Nanomaterial. J. Control. Release.

[72] Rampado R., Crotti S., Caliceti P., Pucciarelli S., Agostini M. (2020). Recent Advances in Understanding the Protein Corona of Nanoparticles and in the Formulation of “Stealthy” Nanomaterials. Front. Bioeng. Biotechnol..

[73] Rosa S., Connolly C., Schettino G., Butterworth K.T., Prise K.M. (2017). Biological Mechanisms of Gold Nanoparticle Radiosensitization. Cancer Nanotechnol..

