# Supplementary material for: Effects of Surface Protein Adsorption on the Distribution and Retention of Intratumorally Administered Gold Nanoparticles
Source: Pharmaceutics. 2021 Feb 5;13(2):216. doi: 10.3390/pharmaceutics13020216 (PMC7914653; doi:10.3390/pharmaceutics13020216)
Supplement: Supplementary file 1 [file pharmaceutics-13-00216-s001.pdf]

pharmaceutics

MDPI

Supplementary Materials: Effects of Surface Protein Adsorption on the Distribution and Retention of Intratumorally Administered Gold Nanoparticles

Rossana Terracciano, Aobo Zhang, E. Brian Butler, Danilo Demarchi, Jason H. Hafner, Alessandro Grattoni and Carly S. Filgueira

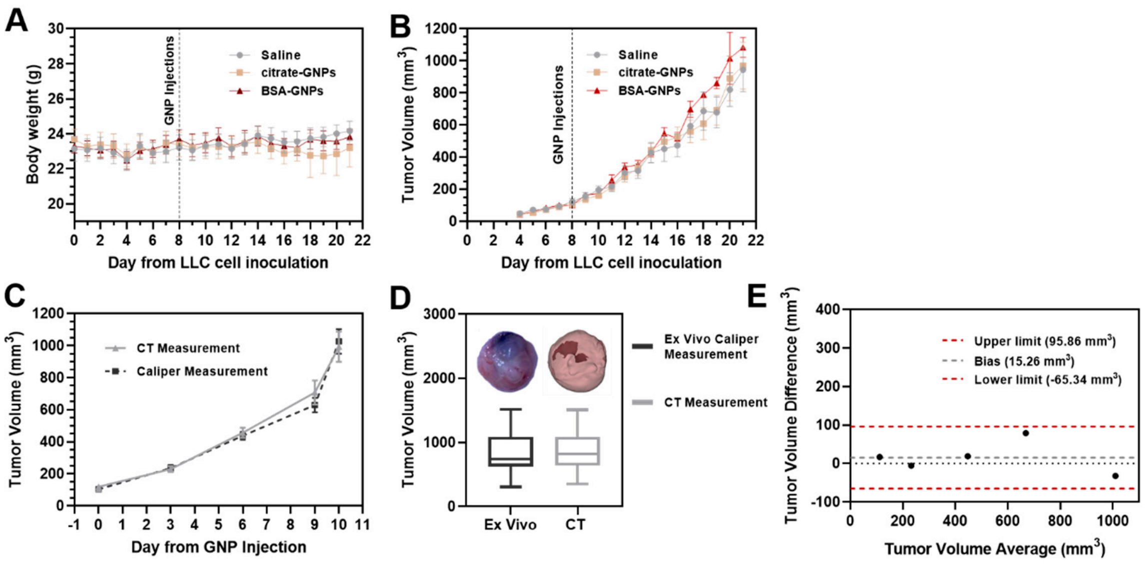

Figure S1. Mice weight and tumor measurements. (**A**) Body weight of female black mice (n = 8/group). (**B**) Tumor volume measured using a caliper (n = 8/group). (**C**) Comparison between tumor volumes measured manually by external caliper or quantified from micro-CT analysis (n = 16 mice for each measurement modality). No significant differences highlighted. Each data point in A–C represents the mean ± standard error of the mean (s.e.m.). (**D**) Data are shown in boxplots for Day 10. (**E**) Bland Altman comparison between tumor volumes measured manually by external caliper or quantified from micro-CT analysis. A bias of 15 mm³ is detected.
